# Anterior–posterior topographic patterns of thoracic joint structural abnormalities on chest CT in psoriatic arthritis

**DOI:** 10.3389/fmed.2026.1944735

**Published:** 2026-08-26

**Authors:** Rui Li, Hui Sang, Yuxiang Zhang, Yong Wang, Yu Gao, Jianzhao Wang, Xiaomeng Qin, Yanfang Song, Tianshu Li

**Affiliations:** 1 Department of Radiology, Hebei Provincial Hospital of Traditional Chinese Medicine, Shijiazhuang, Hebei Province, China; 2 The First Orthopedics Department, Hebei Provincial Hospital of Traditional Chinese Medicine, Shijiazhuang, Hebei Province, China

**Keywords:** computed tomography, costotransverse joint, costovertebral joint, imaging phenotype, psoriatic arthritis, sternoclavicular joint, thoracic ring

## Abstract

**Objective:**

This study sought to develop and evaluate a chest computed tomography (CT)-based thoracic ring topographic framework for characterizing the anterior–posterior distribution of structural involvement in patients with psoriatic arthritis (PsA).

**Methods:**

This retrospective study included 279 patients with PsA who underwent clinically indicated chest CT. The sternoclavicular joint (SCJ), costovertebral joint (CVJ), and costotransverse joint (CTJ) were assessed. A compartment was considered positive when its structural burden score exceeded 0 or any prespecified structural feature was present. Patients were classified as anterior–posterior (A–P) ring, posterior-only, negative, or anterior-only phenotypes.

**Results:**

SCJ, CVJ, and CTJ positivity occurred in 131 (47.0%), 232 (83.2%), and 236 (84.6%) patients, respectively. All SCJ-positive patients were also positive at both posterior compartments, and no anterior-only phenotype was observed. The A–P ring, posterior-only, and negative groups comprised 131 (47.0%), 111 (39.8%), and 37 (13.3%) patients. Median total structural burden was 59.0, 40.0, and 0.0, respectively (*P* < 0.001). After adjustment, A–P ring involvement was associated with higher total burden than posterior-only involvement (*β* = 7.98, 95% CI: 3.42–12.54; *P* < 0.001), whereas the adjusted difference in posterior burden was not statistically significant (*β* = 4.45, 95% CI: −0.06 to 8.97; *P* = 0.053).

**Conclusion:**

SCJ positivity occurred within a combined anterior–posterior pattern and was associated with greater overall structural burden. Thoracic ring topography may provide a spatial framework that requires independent and multimodal validation.

## Introduction

Psoriatic arthritis (PsA) is a heterogeneous inflammatory musculoskeletal disease in which erosive damage, osteolysis, new bone formation, enthesophytes, and ankylosis may coexist (1–3). This mixed remodeling phenotype complicates interpretation because the distribution and meaning of structural lesions vary across anatomical compartments. Radiography remains central for established damage, whereas magnetic resonance imaging and ultrasonography depict active inflammatory and soft-tissue abnormalities. Computed tomography (CT) provides higher spatial resolution for cortical interruption, joint-space change, osseous proliferation, and fusion in complex osseous regions, although radiation exposure precludes its use as a screening examination (2, 3).

The sternoclavicular joint (SCJ), a synovial articulation of the anterior chest wall, is the only true bony connection between the upper limb girdle and axial skeleton. Its involvement is often underrecognized because symptoms are nonspecific and routine radiography incompletely depicts the joint. Clinical SCJ involvement in PsA has been associated with higher swollen-joint counts, enthesitis, and dactylitis (4), while imaging studies show that anterior chest wall abnormalities may involve the SCJ, manubriosternal joint, and sternocostal region even without chest-wall symptoms (5–7). Posteriorly, the ribs articulate with the thoracic spine through the costovertebral joints (CVJs) and, from T1 to T10, the costotransverse joints (CTJs). CT studies in spondyloarthritis have demonstrated erosion, narrowing, osteophytes, syndesmophytes, and ankylosis at these sites, with CVJ/CTJ abnormalities linked to axial damage, reduced chest expansion, impaired mobility, and syndesmophyte progression (8–10). However, new bone formation is not disease-specific and may overlap with degenerative remodeling (11).

A key unresolved question is whether anterior and posterior abnormalities form reproducible spatial patterns. Site-based reporting answers which joints are abnormal, and burden scoring answers how much damage is present, but neither directly states whether anterior and posterior abnormalities form recurring spatial combinations. This distinction matters because identical joint counts may represent different anatomical distributions and potentially different reporting concepts. Anatomically, the SCJ constitutes an anterior thoracic endpoint, whereas the CVJ and CTJ form paired posterior paravertebral endpoints. Integrating these compartments may convert a list of positive joints into an interpretable thoracic ring pattern without replacing quantitative structural scoring.

We developed a chest CT-based thoracic ring topographic framework for opportunistic evaluation of clinically indicated chest CT. The primary aim was to determine whether SCJ structural involvement occurred in isolation or together with posterior CVJ/CTJ involvement and thereby to characterize recurring anterior–posterior (A–P) spatial patterns. Secondary aims were to compare structural burden and feature composition across the resulting topographic phenotypes. Exploratory analyses evaluated adjusted associations, alternative phenotype definitions, follow-up descriptions, and model fit.

## Materials and methods

### Study design and participants

**Figure 1:**
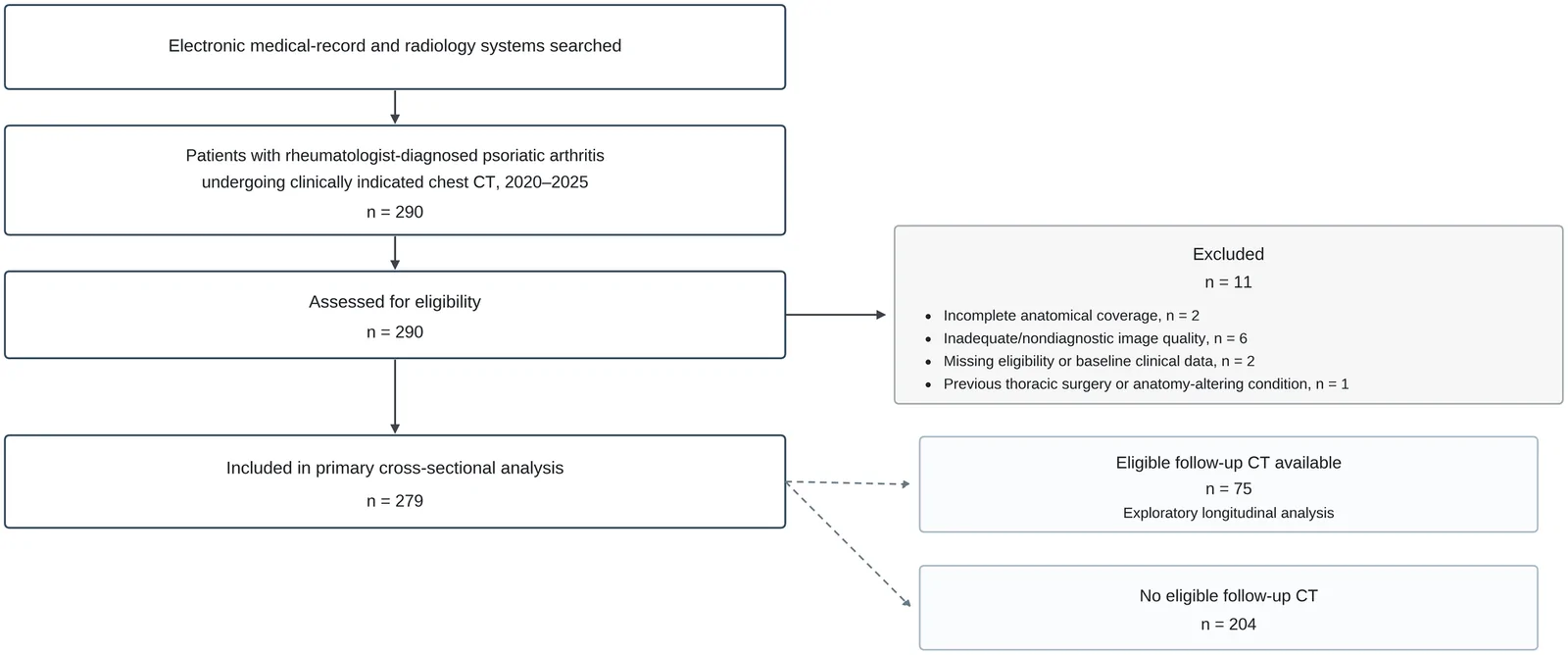
Study flow diagram. Flowchart of patient identification, eligibility assessment, exclusions, inclusion in the primary cross-sectional analysis, and availability of eligible follow-up CT for the exploratory longitudinal analysis. CT, computed tomography.

This retrospective single-center study was approved by the Medical Ethics Committee of Hebei Provincial Hospital of Traditional Chinese Medicine (No. HBZY2026-KY-055-01), and the requirement for written informed consent was waived. The study followed the Declaration of Helsinki and is reported in accordance with the Reporting of Observational Studies in Epidemiology (STROBE) recommendations. Patient identification, eligibility assessment, and cohort selection are summarized in Figure 1. Consecutive patients with rheumatologist-diagnosed PsA who underwent clinically indicated chest CT between January 2020 and December 2025 were identified from institutional records. Classification Criteria for PsA (CASPAR) (12) fulfillment was verified from contemporaneous rheumatology records, independently of the thoracic CT findings.

Patients were eligible if they met all of the following criteria: (1) rheumatologist-diagnosed PsA fulfilling the CASPAR criteria; (2) chest CT coverage of the bilateral SCJs, T1–T12 CVJs, and T1–T10 CTJs; and (3) adequate image quality for assessment of all target compartments. Exclusion criteria were: (1) incomplete coverage or nondiagnostic imaging; (2) insufficient clinical data to verify eligibility or baseline covariates; (3) prior thoracic disease, surgery, or intervention that substantially altered the relevant anatomy.

For patients with multiple eligible chest CT examinations, the earliest scan obtained on or after the documented PsA diagnosis was selected as baseline. If no postdiagnosis scan was available, a CT performed within 1 month before diagnosis was accepted only when contemporaneous records confirmed ongoing PsA symptoms and diagnostic evaluation. Follow-up examinations were those obtained after baseline and on or before June 30, 2026; the last eligible scan was used for exploratory longitudinal comparison, provided that the interval from baseline was at least 2 months. Baseline phenotype was defined using the baseline CT only and was not revised using follow-up findings.

### Clinical variables

Clinical characteristics and laboratory findings were obtained through retrospective review of the medical records. Disease duration was defined as the interval between the documented date of symptom onset or PsA diagnosis, whichever was available in the clinical record, and the date of the baseline CT examination. Disease duration was used as a clinical descriptor and covariate but was not used to assign patients to structural disease phases, because the cross-sectional CT findings reflect cumulative structural damage rather than a validated temporal stage of PsA. Baseline was defined as the date of the baseline CT used for analysis. Laboratory values were taken from the measurement closest to CT within ±7 days. Disease duration was calculated from symptom onset or diagnosis to CT. Standard laboratory cut-offs were applied as described. Psoriasis area and severity index (PASI) were inconsistently recorded (46.6%, 129/277) and not obtained within a standardized interval to CT; therefore, it was not analyzed as a baseline variable. Current psoriasis status could not be reliably determined and was not included as a binary variable. Composite PsA activity scores were not calculated due to missing components, and other standardized clinical assessments were unavailable.

The following thresholds were applied to adult patients: antistreptolysin O (ASO) >200 IU/mL, procalcitonin (PCT) ≥0.25 ng/mL, C-reactive protein (CRP) >10 mg/L, and rheumatoid factor (RF) >20 IU/mL. Antinuclear antibody (ANA) status was categorized as positive according to the antibody titer reported by the laboratory. An elevated erythrocyte sedimentation rate (ESR) was defined using the corresponding age- and sex-specific reference limits.

Composite measures of PsA activity, including the Disease Activity Index for Psoriatic Arthritis (DAPSA) and the Psoriatic Arthritis Disease Activity Score (PASDAS), were not calculated due to incomplete data within the required time window around baseline CT. Standardized assessments of chest expansion, chest-wall pain, patient-reported outcomes, and standardized longitudinal clinical outcomes were unavailable and excluded.

### CT acquisition and reconstruction

Routine multidetector platforms included GE Revolution, United Imaging 80-row, and Siemens 64-row CT. Patients were supine with arms raised when feasible, and coverage extended from the thoracic inlet through the costophrenic angles. Protocols were centered on 120 kVp with automatic tube-current or exposure control. Standard chest and high-spatial-frequency bone reconstructions were generated. Review used 1.0-mm axial, coronal, and sagittal bone images at 1.0-mm or overlapping intervals (window width/level 2,000/500 HU, Supplementary Table S1).

### Image review and reader reliability

Two radiologists with 8 and 10 years of relevant CT experience independently reviewed all examinations after standardized training with a written scoring atlas and 20 nonstudy calibration cases. Bilateral SCJs, T1–T12 CVJs, and T1–T10 CTJs were assessed on multiplanar bone-window images. Readers were blinded to clinical data, the other reader's scores, and derived phenotypes, and recorded all features and severity grades before consensus review. Disagreements were resolved by joint review. Consensus ratings were used for phenotype and burden analyses, whereas independent pre-consensus ratings were used to assess reliability with percentage agreement, Cohen's kappa for binary or categorical variables, and intraclass correlation coefficient (ICC) with 95% confidence intervals for continuous burden scores (Supplementary Table S2).

### CT structural feature definitions

Erosion was focal cortical interruption or irregular articular bone loss. Narrowing was definite joint-space reduction, irregularity, or loss. Osteophytes were marginal or periarticular osseous outgrowths. Sclerosis was definite increased subchondral density or remodeling adjacent to the articular surface. Ankylosis was partial or complete osseous bridging across the joint. Each feature was coded independently, allowing destructive, proliferative, and locking abnormalities to coexist in the same compartment.

### Joint-compartment positivity and structural burden scoring

Each assessable joint was graded from 0 to 4 according to the overall severity of CT-detectable structural abnormality. Grade 0 indicated a normal joint without structural abnormality. Grade 1 indicated a suspicious structural abnormality. Grade 2 indicated mild structural abnormality, such as focal subtle erosion, sclerosis, or slight joint-space change. Grade 3 indicated moderate structural abnormality, such as definite subchondral bone destruction, marked joint-space narrowing, or articular irregularity. Grade 4 indicated severe structural abnormality or complete ankylosis, characterized by osseous bridging across the joint space. The same semiquantitative grading principle was applied to the SCJ, CVJ, and CTJ. Representative CT examples illustrating the practical application of the severity grades are provided in Supplementary Figure S1.

Left and right scores were summed within each anatomical compartment, yielding possible ranges of 0–8 for the SCJ, 0–96 for the CVJ, and 0–80 for the CTJ. Total structural burden was calculated as the sum of the SCJ, CVJ, and CTJ scores, whereas posterior structural burden was calculated as the sum of the CVJ and CTJ scores.

Under the primary broad definition, a target compartment was classified as positive if the bilateral structural burden score was >0 or if any prespecified structural feature was present. This definition intentionally included grade-1 suspicious abnormalities to provide a sensitivity-oriented characterization of CT-detectable structural involvement. We recognized that this approach may reduce specificity because some compartments could be classified as positive on the basis of subtle or equivocal findings; this potential effect on phenotype classification was therefore considered in the interpretation of the topographic analyses. In the sensitivity analysis, positivity was defined using partial or complete ankylosis only. This substantially more stringent definition was used to examine the dependence of topographic classification on the threshold used to define structural involvement and was not adopted as the primary definition because strict SCJ ankylosis was uncommon.

### Thoracic ring topographic phenotypes

Four mutually exclusive phenotypes were predefined: A–P ring involvement, defined as SCJ positivity together with positivity of the CVJ or CTJ; posterior-only, defined as SCJ negativity with positivity of the CVJ or CTJ; anterior-only involvement, defined as SCJ positivity with negativity of both the CVJ and CTJ; and negative involvement, defined as negativity at all three joint sites. In the sensitivity analysis, positivity was defined as ankylosis and was not used primarily because strict SCJ ankylosis was rare.

### Study outcomes and exploratory analyses

The primary outcome was the distribution of CT-positive target compartments and topographic phenotypes, including anterior-only involvement. Total structural burden was the principal quantitative outcome, while posterior burden (CVJ + CTJ) was analyzed to reduce direct coupling with SCJ-based phenotype classification. Secondary outcomes included compartment scores, the number of positive compartments, patient-level structural features, and adjusted phenotype–burden associations. Exploratory analyses included ankylosis-based phenotypes, alternative feature counts, model comparisons, and longitudinal change.

For follow-up, baseline and final CT examinations were scored using identical criteria; progression was defined as an increase of at least 1 point accompanied by a newly involved joint or definite structural feature, improvement as a decrease of at least 1 points without new lesions, and all other cases as unchanged. Follow-up duration was summarized as the median and interquartile range, and longitudinal findings were considered exploratory because scan timing and indications were nonstandardized.

### Statistical analysis

Continuous variables were summarized as medians with interquartile ranges (IQRs), whereas categorical variables were presented as counts and percentages, *n* (%), using nonmissing observations as the denominators. Differences among the three observed phenotypes were assessed using the Kruskal–Wallis test for continuous variables and the chi-square test or Fisher's exact test, as appropriate, for categorical variables. Pairwise comparisons of imaging burden were performed using the Mann–Whitney *U* test with Bonferroni correction for multiple testing. Associations between individual features and A–P ring vs. posterior-onlys were expressed as odds ratios (ORs) with 95% confidence intervals (CIs), together with *P* values derived from Fisher's exact test.

Multivariable linear regression models were fitted using heteroskedasticity-consistent type 3 (HC3) robust standard errors and were adjusted for age, sex, disease duration, elevated CRP, elevated ESR, and phenotype indicators. Direct contrasts between the A–P ring and posterior-only were estimated from the fitted models. Exploratory measures of model performance included the coefficient of determination (*R*^2^), adjusted coefficient of determination (adjusted *R*^2^), Akaike information criterion (AIC), Bayesian information criterion (BIC), and root mean square error (RMSE). All analyses were conducted using Python version 3.11.7 and prespecified reproducible scripts. Statistical tests were two-sided, with *P* < 0.05 considered the conventional threshold for statistical significance. Borderline findings were reported without being interpreted as evidence of equivalence.

## Results

### Patient characteristics

A total of 279 patients (837 compartment-level records) were included (Table 1). Median age was 40.0 years and differed across groups (52.0, 37.0, and 20.0 years for A–P, posterior-only, and negative; *P* < 0.001). Disease duration also differed (10.0, 8.0, and 4.0 years; *P* = 0.004). Sex distribution was similar (male: 73.3%, 78.4%, and 73.0%; *P* = 0.619). Elevated CRP occurred in 8.4%, 1.8%, and 2.7% of the three groups (*P* = 0.051), and elevated ESR in 6.1%, 3.6%, and 5.4% (*P* = 0.669). Other available baseline laboratory characteristics, including ASO, PCT, RF, and ANA status, as well as biologic therapy use, are summarized by topographic phenotype in Table 1.

**Table 1:** Baseline characteristics of the study cohort by thoracic ring topographic phenotype.

Characteristic	Overall (*N* = 279)	A–P ring type (*n* = 131)	posterior-only type (*n* = 111)	Negative type (*n* = 37)	*P* value
Age, years	40.0 (32.0–54.0)	52.0 (39.5–61.5)	37.0 (32.0–46.5)	20.0 (16.0–26.0)	<0.001
Disease duration, years	8.0 (2.0–16.0)	10.0 (3.0–20.0)	8.0 (2.0–11.0)	4.0 (1.0–8.0)	0.004
Male sex	210 (75.3%)	96 (73.3%)	87 (78.4%)	27 (73.0%)	0.619
Elevated CRP	14/279 (5.0%)	11/131 (8.4%)	2/111 (1.8%)	1/37 (2.7%)	0.062
Elevated ESR	14/279 (5.0%)	8/131 (6.1%)	4/111 (3.6%)	2/37 (5.4%)	0.689
Elevated ASO	7 (2.5)	2 (1.5)	3 (2.7)	2 (5.4)	0.354
Elevated PCT	26 (9.3)	17 (13.0)	4 (3.6)	5 (13.5)	0.016
RF positive	1 (0.4)	0 (0.0)	1 (0.9)	0 (0.0)	0.530
ANA positive	50 (17.9)	32 (24.4)	13 (11.7)	5 (13.5)	0.028
Biologic therapy use	127/279 (45.5%)	55/131 (42.0%)	55/111 (49.5%)	17/37 (45.9%)	0.499

Values are median (interquartile range) or *n* (%), unless otherwise indicated. Baseline refers to the date of the baseline CT examination used for the primary cross-sectional analysis. Laboratory variables were based on the measurement obtained closest to the baseline CT examination within 7 days before or after CT. *P* values for continuous variables were calculated using the Kruskal–Wallis test. Pearson's *χ*^2^ test was used for categorical variables when expected cell counts were adequate; the Fisher–Freeman–Halton exact test was used for sparse contingency tables. A–P, anterior–posterior; ANA, antinuclear antibody; ASO, antistreptolysin O; CRP, C-reactive protein; CT, computed tomography; ESR, erythrocyte sedimentation rate; PCT, procalcitonin; RF, rheumatoid factor.

### Distribution of CT positivity across target joints

The distribution of CT-positive target joints differed across the SCJ, CVJ, and CTJ compartments (Table 2). SCJ positivity was detected in 131 of 279 patients (47.0%), whereas CVJ and CTJ positivity were detected in 232 patients (83.2%) and 236 patients (84.6%), respectively.

**Table 2:** Distribution of CT-positive target joints, joint-positive combinations, and thoracic ring topographic phenotypes.

Module	Measure	Patients or numerator	Denominator	Percentage	Definition or interpretation
Cohort	Final analyzable patients	279	—	—	Final analyzable sample in the cleaned main-analysis dataset
Cohort	Main-analysis joint records	837	—	—	Patients×3 target joints
Joint positivity	SCJ positive	131	279	47.0%	Broad CT structural positivity
Joint positivity	CVJ positive	232	279	83.2%	Broad CT structural positivity
Joint positivity	CTJ positive	236	279	84.6%	Broad CT structural positivity
Positive joint combination	SCJ + CVJ + CTJ all positive	131	279	47.0%	Three-joint SCJ/CVJ/CTJ combination
Positive joint combination	SCJ + CVJ positive without CTJ	0	279	0.0%	Three-joint SCJ/CVJ/CTJ combination
Positive joint combination	SCJ + CTJ positive without CVJ	0	279	0.0%	Three-joint SCJ/CVJ/CTJ combination
Positive joint combination	Isolated SCJ positive	0	279	0.0%	Three-joint SCJ/CVJ/CTJ combination
Positive joint combination	CVJ + CTJ positive without SCJ	95	279	34.1%	Three-joint SCJ/CVJ/CTJ combination
Positive joint combination	Isolated CVJ positive	6	279	2.2%	Three-joint SCJ/CVJ/CTJ combination
Positive joint combination	Isolated CTJ positive	10	279	3.6%	Three-joint SCJ/CVJ/CTJ combination
Positive joint combination	All three target joints negative	37	279	13.3%	Three-joint SCJ/CVJ/CTJ combination
Topographic phenotype	Anterior-posterior ring type	131	279	47.0%	Defined by the anterior SCJ and posterior CVJ/CTJ combination
Topographic phenotype	posterior-only type	111	279	39.8%	Defined by the anterior SCJ and posterior CVJ/CTJ combination
Topographic phenotype	Negative type	37	279	13.3%	Defined by the anterior SCJ and posterior CVJ/CTJ combination
Topographic phenotype	anterior-only	0	279	0.0%	Defined by the anterior SCJ and posterior CVJ/CTJ combination

Broad CT structural positivity was defined by a positive joint-level structural involvement field in the cleaned dataset, based on left/right score >0 or any positive structural CT sign.

The anterior-only was predefined as SCJ positivity without CVJ/CTJ positivity but had 0 patients in the current dataset. Percentages are calculated using *N* = 279.

SCJ, sternoclavicular joint; CVJ, costovertebral joint; CTJ, costotransverse joint.

All 131 SCJ-positive patients were also positive at both posterior compartments, forming an SCJ + CVJ + CTJ pattern. No patient had SCJ + CVJ positivity without CTJ positivity, SCJ + CTJ positivity without CVJ positivity, or isolated SCJ positivity. Among SCJ-negative patients, 95 patients (34.1% of the overall cohort) were positive at both posterior compartments, 6 (2.2%) had isolated CVJ positivity, and 10 (3.6%) had isolated CTJ positivity. All three target compartments were negative in 37 patients (13.3%).

### Thoracic ring topographic phenotypes

The predefined topographic classification identified three observed thoracic ring phenotypes (Table 2). The A–P ring phenotype was present in 131 patients (47.0%), the posterior-only in 111 patients (39.8%), and the negative phenotype in 37 patients (13.3%). The predefined anterior-only phenotype was not observed in the primary analysis. Although the minimum definition of the A–P ring phenotype required SCJ positivity together with positivity of either the CVJ or CTJ, all patients classified into this group were positive at all three compartments.

### Structural burden across topographic phenotypes

**Figure 2:**
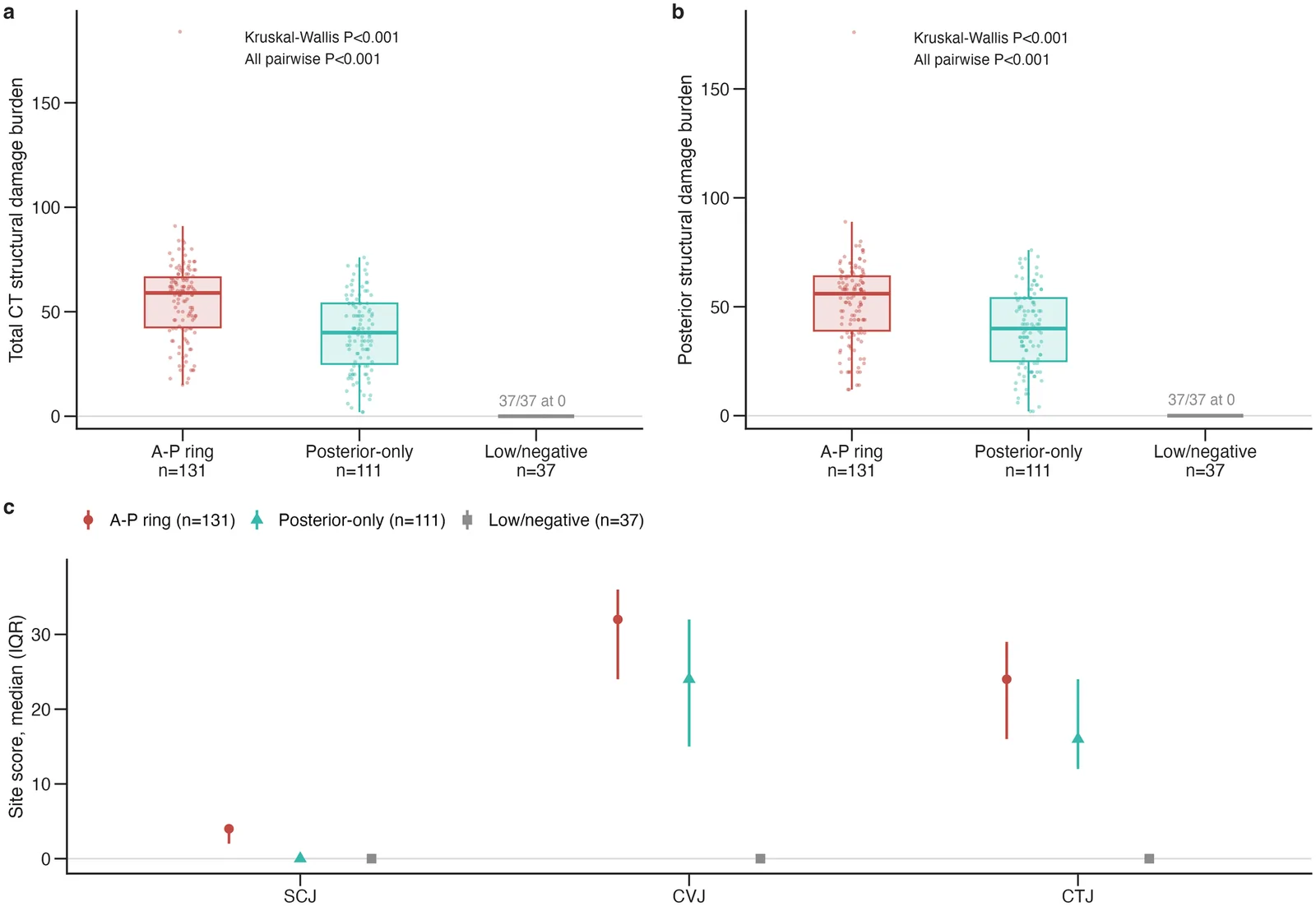
CT structural burden across thoracic ring topographic phenotypes. **(a)** Total CT structural burden; **(b)** posterior structural burden; and **(c)** site-specific SCJ, CVJ, and CTJ scores in the anterior–posterior (A–P) ring, posterior-only, and negative phenotypes. Boxplots show medians and interquartile ranges with individual observations; points and error bars show medians and interquartile ranges. CTJ, costotransverse joint; CVJ, costovertebral joint; SCJ, sternoclavicular joint.

CT structural burden differed significantly across the three phenotypes (Table 3; Figure 2). Median total scores were 59.0 (IQR: 42.5–66.5) in the A–P ring group, 40.0 (IQR: 25.0–54.0) in the posterior-only group, and 0.0 (IQR: 0.0–0.0) in the negative group (*P* < 0.001), with all pairwise comparisons significant (*P* < 0.001). Posterior structural burden showed a similar pattern, with median scores of 56.0 (IQR: 39.0–64.0), 40.0 (IQR: 25.0–54.0), and 0.0 (IQR: 0.0–0.0), respectively (*P* < 0.001). Median SCJ scores were 4.0, 0.0, and 0.0; CVJ scores were 32.0, 24.0, and 0.0; and CTJ scores were 24.0, 16.0, and 0.0 across the three groups. The median number of positive compartments was 3.0, 2.0, and 0.0, respectively (all *P* < 0.001).

**Table 3:** Structural damage burden by thoracic ring topographic phenotype.

Outcome	Overall (*N* = 279)	A–P ring type (*n* = 131)	posterior-only type (*n* = 111)	Negative type (*n* = 37)	Overall *P* value	A–P vs. posterior-only	A–P vs. Negative	posterior-only vs. Negative
Total CT structural damage burden	46.0 (24.0–61.0)	59.0 (42.5–66.5)	40.0 (25.0–54.0)	0.0 (0.0–0.0)	<0.001	<0.001	<0.001	<0.001
Posterior structural damage burden	44.0 (22.0–58.0)	56.0 (39.0–64.0)	40.0 (25.0–54.0)	0.0 (0.0–0.0)	<0.001	<0.001	<0.001	<0.001
SCJ score	0.0 (0.0–4.0)	4.0 (2.0–4.0)	0.0 (0.0–0.0)	0.0 (0.0–0.0)	<0.001	<0.001	<0.001	1.000
CVJ score	26.0 (12.0–34.0)	32.0 (24.0–36.0)	24.0 (15.0–32.0)	0.0 (0.0–0.0)	<0.001	<0.001	<0.001	<0.001
CTJ score	18.0 (10.0–26.0)	24.0 (16.0–29.0)	16.0 (12.0–24.0)	0.0 (0.0–0.0)	<0.001	<0.001	<0.001	<0.001
Number of positive target joints	2.0 (2.0–3.0)	3.0 (3.0–3.0)	2.0 (2.0–2.0)	0.0 (0.0–0.0)	<0.001	<0.001	<0.001	<0.001

Values are median (interquartile range). Overall *P* values were calculated using Kruskal–Wallis tests.

Pairwise *P* values are Bonferroni-corrected Mann–Whitney *U* tests. Total CT structural damage burden equals SCJ + CVJ + CTJ scores; posterior structural damage burden equals CVJ + CTJ scores.

A–P, anterior–posterior; SCJ, sternoclavicular joint; CVJ, costovertebral joint; CTJ, costotransverse joint.

### CT structural-feature profiles

**Figure 3:**
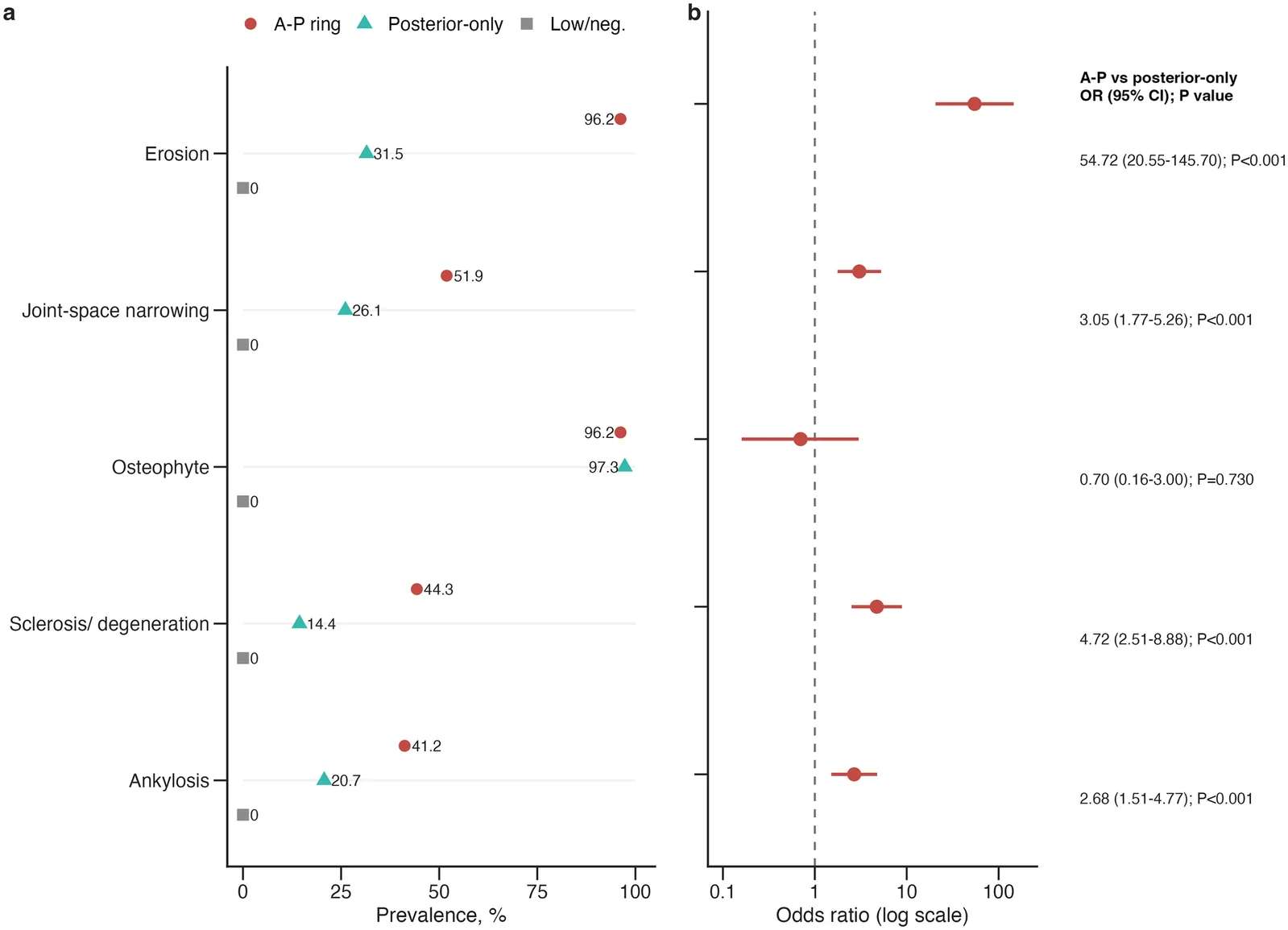
CT structural-feature profiles across thoracic ring topographic phenotypes. **(a)** Prevalence of structural CT features in the A–P ring, posterior-only, and negative phenotypes. **(b)** Odds ratios and 95% confidence intervals comparing the A–P ring with the posterior-only phenotype. The dashed vertical line indicates an odds ratio of 1. A–P, anterior–posterior; CI, confidence interval; OR, odds ratio.

Patient-level CT structural-feature profiles differed between the A–P ring and posterior-only (Table 4; Figure 3). Compared with patients with posterior-only involvement, those with A–P ring involvement had higher frequencies of erosion [126/131 (96.2%) vs. 35/111 (31.5%); OR = 54.72, 95% CI: 20.55–145.70; *P* < 0.001], joint-space narrowing [68/131 (51.9%) vs. 29/111 (26.1%); OR = 3.05, 95% CI: 1.77–5.26; *P* < 0.001], sclerosis or degenerative change [58/131 (44.3%) vs. 16/111 (14.4%); OR = 4.72, 95% CI: 2.51–8.88; *P* < 0.001], and partial or complete ankylosis [54/131 (41.2%) vs. 23/111 (20.7%); OR = 2.68, 95% CI: 1.51–4.77; *P* < 0.001].

**Table 4:** CT structural features by thoracic ring topographic phenotype and anterior-posterior vs. posterior-only associations.

CT structural feature	Overall (*N* = 279)	A–P ring type (*n* = 131)	posterior-only type (*n* = 111)	Negative type (*n* = 37)	Overall *P* value	A–P vs. posterior-only OR (95% CI)	A–P vs. posterior-only *P* value
Any erosion	161 (57.7%)	126 (96.2%)	35 (31.5%)	0 (0.0%)	<0.001	54.72 (20.55–145.70)	<0.001
Any joint-space narrowing	97 (34.8%)	68 (51.9%)	29 (26.1%)	0 (0.0%)	<0.001	3.05 (1.77–5.26)	<0.001
Any osteophyte	234 (83.9%)	126 (96.2%)	108 (97.3%)	0 (0.0%)	<0.001	0.70 (0.16–3.00)	0.730
Any sclerosis/degenerative change	74 (26.5%)	58 (44.3%)	16 (14.4%)	0 (0.0%)	<0.001	4.72 (2.51–8.88)	<0.001
Any partial or complete bony ankylosis	77 (27.6%)	54 (41.2%)	23 (20.7%)	0 (0.0%)	<0.001	2.68 (1.51–4.77)	<0.001

Values are *n* (%). Overall *P* values were calculated using chi-square tests.

Odds ratios compare the anterior-posterior ring type with the posterior-only type; Fisher exact tests were used for the two-group comparison.

A–P, anterior–posterior; CI, confidence interval; CT, computed tomography; OR, odds ratio.

Osteophyte formation was highly prevalent in both groups, occurring in 126 of 131 patients (96.2%) with A–P ring involvement and 108 of 111 patients (97.3%) with posterior-only involvement. Osteophyte formation did not significantly distinguish between the two phenotypes (OR = 0.70, 95% CI: 0.16–3.00; *P* = 0.730). None of the 37 patients in the negative group had any of the five patient-level structural features under the primary coding rule.

### Association between topographic phenotype and structural burden

After adjustment for age, sex, disease duration, elevated CRP, and ESR, both the A–P ring and posterior-only were associated with higher total structural burden than the negative phenotype (Table 5). Compared with the negative group, adjusted coefficients were *β* = 33.87 (95% CI: 27.87–39.88; *P* < 0.001) for the A–P ring phenotype and *β* = 25.89 (95% CI: 21.48–30.31; *P* < 0.001) for the posterior-only. The A–P ring phenotype also showed higher total burden than the posterior-only (*β* = 7.98, 95% CI: 3.42–12.54; *P* < 0.001).

**Table 5:** Multivariable association of thoracic ring topographic phenotype with structural damage burden.

Outcome	Model contrast/exposure	Adjusted *β*, points	95% CI	*P* value	*N*	*R* ^2^	Adjusted *R*^2^
Total CT structural damage burden	Anterior-posterior ring type vs. Negative type	33.87	27.87–39.88	<0.001	279	0.626	0.617
Total CT structural damage burden	posterior-only type vs. Negative type	25.89	21.48–30.31	<0.001	279	0.626	0.617
Total CT structural damage burden	Anterior-posterior ring type vs. posterior-only type	7.98	3.42–12.54	<0.001	279	0.626	0.617
Posterior structural damage burden	Anterior-posterior ring type vs. Negative type	30.51	24.55–36.46	<0.001	279	0.604	0.594
Posterior structural damage burden	posterior-only type vs. Negative type	26.05	21.64–30.46	<0.001	279	0.604	0.594
Posterior structural damage burden	Anterior-posterior ring type vs. posterior-only type	4.45	−0.06–8.97	0.053	279	0.604	0.594

Adjusted beta coefficients were estimated using multivariable linear models with HC3 robust standard errors.

Each model adjusted for age, sex, disease duration, elevated CRP, and elevated ESR. Negative type was the reference for rows comparing each phenotype with the reference group; direct anterior-posterior vs. posterior-only contrasts are also shown.

*R*^2^ and adjusted *R*^2^ refer to the fitted model for the corresponding outcome. A–P, anterior–posterior; CI, confidence interval; CRP, C-reactive protein; ESR, erythrocyte sedimentation rate.

For posterior structural burden, adjusted coefficients vs. the negative phenotype were *β* = 30.51 (95% CI: 24.55–36.46; *P* < 0.001) for the A–P ring phenotype and *β* = 26.05 (95% CI: 21.64–30.46; *P* < 0.001) for the posterior-only. The A–P ring vs. posterior-only comparison was positive but not statistically significant (*β* = 4.45, 95% CI: −0.06 to 8.97; *P* = 0.053).

### Sensitivity and exploratory analyses

**Figure 4:**
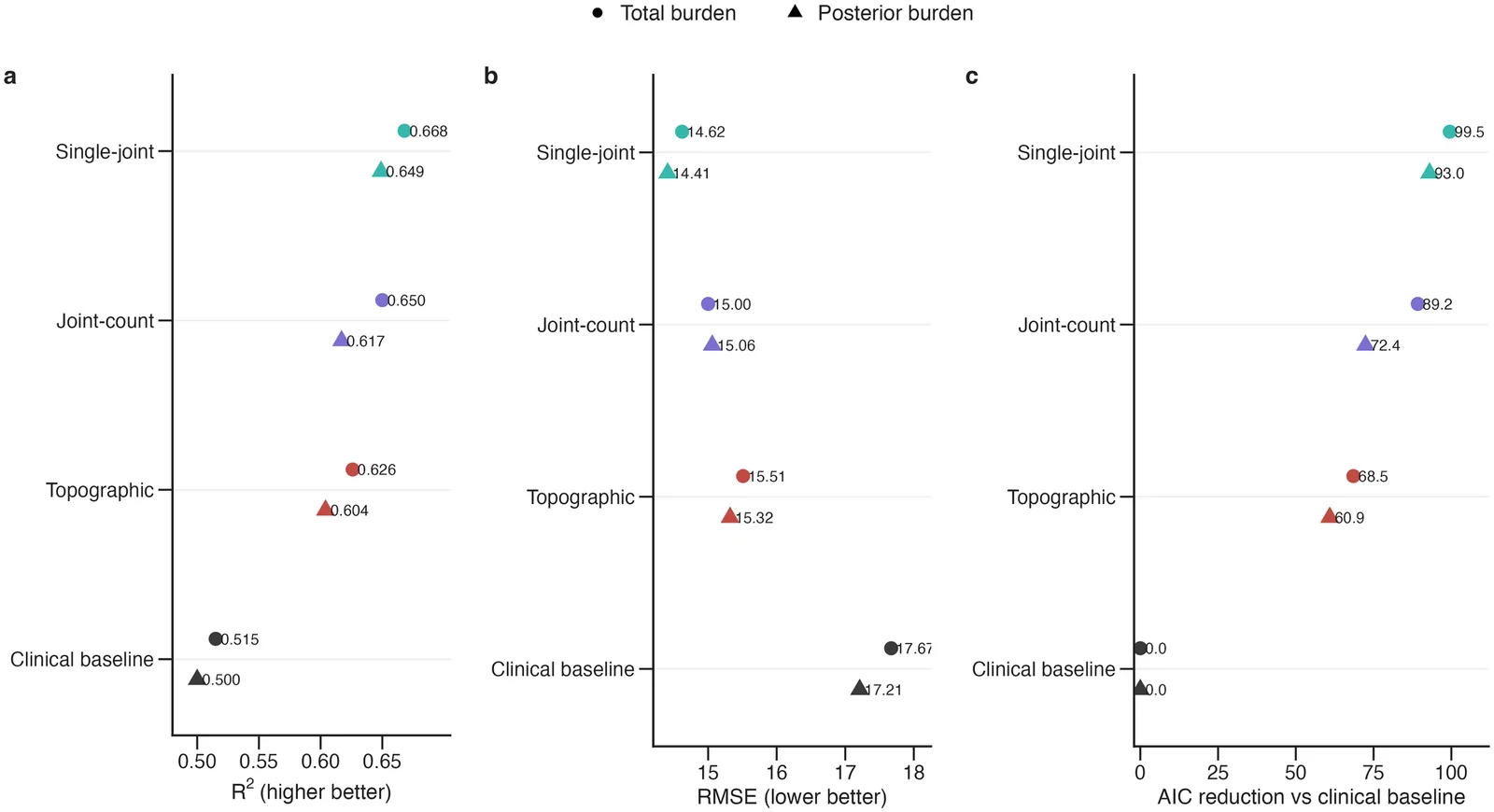
Comparative performance of models for CT structural burden. Model performance for total and posterior structural burden according to **(a)** *R*^2^, **(b)** root mean square error, and **(c)** Akaike information criterion reduction relative to the clinical baseline model. The clinical baseline, topographic, joint-count, and single-joint models are compared. Circles indicate total burden and triangles indicate posterior burden. AIC, Akaike information criterion; RMSE, root mean square error.

Follow-up data (*n* = 75) showed structural progression in 10/36 A–P, 4/29 posterior-only, and 0/10 negative patients (Supplementary Table S3); findings were exploratory due to limited and non-standardized follow-up. Sensitivity analyses using an ankylosis-only definition classified 2 patients as strict A–P, 72 as posterior-only, 3 as anterior-only, and 202 as having no ankylosis (Supplementary Table S4), differing from the primary definition; structural-feature counts also varied across phenotypes (*P* < 0.001). Inter-reader agreement was high, with agreement rates of 96.1%–99.6%, *κ* values of 0.793–0.920, and intraclass correlation coefficients of 0.935–0.972. Including topographic phenotype improved model fit for total (Δ*R*^2^ = 0.111; ΔAIC = −68.5) and posterior burden (Δ*R*^2^ = 0.104; ΔAIC = −60.9), although joint-based models performed better (Supplementary Table S5; Figure 4).

## Discussion

The principal finding of this study was that SCJ structural positivity did not occur as an isolated anterior abnormality under the primary CT definition. Instead, every SCJ-positive patient also had abnormalities at both the CVJ and CTJ, forming a three-compartment anterior–posterior pattern. This A–P ring phenotype was associated with a greater overall structural burden than posterior-only involvement, even after adjustment for clinical and inflammatory variables. The corresponding difference in posterior burden was smaller and statistically uncertain, however, indicating that the main association was strongest for total rather than for posterior damage independent of the SCJ component. Taken together, these findings support thoracic ring topography as a way of organizing the spatial distribution of CT abnormalities, rather than as a substitute for quantitative burden assessment. The principal contribution of this study is therefore not the identification of individual thoracic joint abnormalities, but the integration of anterior and posterior joint involvement into a spatial topographic framework that complements conventional site-based assessment and quantitative burden scoring.

The spatial framework is based on anatomical compartmentalization rather than an assumed pathway of disease spread. The SCJ was considered the anterior thoracic endpoint, whereas the CVJ and CTJ represented posterior paravertebral compartments. By combining compartment-level CT positivity across these anatomically separated sites, individual joint findings were reorganized into anterior–posterior distribution patterns. Thus, the framework d11escribes where structural abnormalities coexist within the thorax rather than implying that disease progresses from one compartment to another. The biological mechanisms underlying the observed anterior–posterior clustering remain uncertain and require longitudinal and multimodal investigation. The spatial relationship between anterior and posterior involvement extends previous observations concerning the clinical and imaging relevance of the SCJ. Clinical SCJ involvement in PsA has been associated with a greater peripheral disease burden, enthesitis, and dactylitis (4), while CT, MRI, and ultrasonography studies have increasingly recognized anterior chest-wall abnormalities across the spondyloarthritis spectrum (5–7). Those studies established that anterior thoracic joints may be affected even when chest-wall symptoms are absent. They did not, however, determine whether anterior abnormalities usually occur alone or as part of a broader thoracic distribution. In the present cohort, SCJ positivity identified a more extensive pattern that also involved both posterior compartments. This observation should remain population- and definition-specific: the absence of an anterior-only phenotype in the primary analysis does not establish that isolated SCJ disease cannot occur in PsA or in other clinical settings.

Several mechanisms might account for the observed anterior–posterior clustering. One possibility is that the A–P pattern reflects a more extensive inflammatory or structural-remodeling phenotype in which anatomically separated thoracic joints become involved as disease accumulates. The older age and longer disease duration of the A–P group are consistent with cumulative damage as one contributing explanation, although adjustment for these factors did not eliminate the difference in total burden. A second possibility relates to the integrated mechanics of the thoracic cage. The SCJ and anterior rib attachments participate in force transmission at the front of the thorax, whereas the CVJs and CTJs connect the ribs to the spine posteriorly. Biomechanical evidence indicates that the rib cage and thoracic spine function as a coupled system: anterior costosternal connections contribute to global thoracic stability, while the posterior rib articulations reinforce individual spinal segments (13). Restricted motion or altered load transfer in one region could therefore influence stress elsewhere. A third explanation is that the pattern reflects shared exposure to systemic inflammation rather than a direct mechanical sequence. The present cross-sectional data cannot distinguish among these mechanisms, and they do not establish the direction in which anterior and posterior abnormalities develop.

The structural-burden findings also require attention to how the phenotype and outcome were constructed. A–P ring involvement was partly defined by SCJ positivity, while the total burden score included the SCJ component. Some separation between the A–P and posterior-only groups is therefore mathematically expected. The posterior burden score provided a more conservative comparison because it excluded the SCJ contribution. Although posterior burden was higher in the A–P group in the unadjusted analysis, the adjusted difference was smaller and narrowly missed the conventional significance threshold. The findings consequently support an association between A–P involvement and greater overall thoracic structural burden, but only a directionally consistent and less certain association with additional posterior damage. They should not be interpreted as evidence that anterior involvement independently causes progression at the CVJ or CTJ.

Differences in lesion composition add biological and interpretive context to the burden results. Erosion, joint-space narrowing, sclerosis or degenerative change, and ankylosis were all more common in the A–P group than in the posterior-only group. In contrast, osteophytes were nearly universal in both positive phenotypes and offered little discriminatory value. Erosion, narrowing, and ankylosis are recognized components of inflammatory structural damage, whereas sclerosis and new bone formation may arise in both inflammatory and degenerative settings (11, 14). The high prevalence of osteophytes in both groups may therefore represent a background of proliferative or age-related remodeling that is insufficient, when considered alone, to define the broader A–P phenotype. Combinations that include destructive and ankylotic or fusion-related abnormalities may be more informative than isolated proliferative change.

The definition of joint positivity should be considered when interpreting phenotype distribution. The primary rule included grade-1 suspicious abnormalities to favor sensitivity, but this may reduce specificity and introduce potential misclassification, thereby influencing the estimated prevalence of A–P, posterior-only, anterior-only, and negative phenotypes. This issue is relevant in musculoskeletal imaging, where subtle structural changes may overlap with degenerative or mechanical findings, highlighting the need for cautious interpretation of borderline abnormalities (14). More broadly, imaging-based classification depends on the operational definition of positivity. Prior studies in spondyloarthritis have shown that different thresholds and reader interpretation can meaningfully alter the classification of positive findings and overall diagnostic categorization (15, 16). In this study, sensitivity analysis demonstrated this dependence: no anterior-only cases were identified under the primary rule, whereas three were observed under the ankylosis-only definition. These differences reflect that the two definitions capture distinct stages of structural damage. Accordingly, phenotype prevalence should be interpreted as definition-dependent rather than representing fixed biological categories. Future work should evaluate alternative thresholds and their clinical relevance, while standardized definitions and scoring systems may improve reproducibility (17).

Topography and structural burden answer related but different questions. A burden score quantifies the extent of structural abnormality, whereas a topographic label summarizes how positive compartments are arranged. The better numerical fit of the single-joint and positive-joint-count models is therefore unsurprising: those models retain more direct information about the number and location of abnormal sites. The thoracic ring framework should not be presented as the best predictive specification based on the current data. The potential clinical value of the proposed framework is primarily pragmatic and opportunistic. When chest CT has already been acquired for clinical reasons, systematic assessment of the SCJ, CVJ, and CTJ may provide additional musculoskeletal information without recommending CT as a screening examination (18). Rather than reporting abnormalities at these joints as unrelated findings, radiologists could describe whether involvement is posterior-only or extends across the anterior and posterior thoracic compartments, together with the individual structural features and overall burden. Such a structured description may improve recognition of otherwise underreported thoracic joint involvement and facilitate communication between radiologists and rheumatologists. In patients with more extensive thoracic involvement, these findings may also prompt targeted clinical assessment of chest-wall symptoms, functional limitation, and overall musculoskeletal disease status. However, the proposed framework should not currently be interpreted as a validated diagnostic, prognostic, or treatment-decision tool, and prospective controlled studies are required before formal clinical implementation. Such terminology would be consistent with broader efforts to improve standardization, anatomical precision, and clinical communication in spondyloarthritis imaging reports (19, 20). Clinical implementation, however, will require evidence that the terminology is reproducible, understandable, and useful to referring clinicians. Importantly, the proposed topographic phenotypes should not be interpreted as sequential disease stages. They represent cross-sectional patterns of accumulated CT-detectable structural damage, and the present data do not establish a temporal progression from one phenotype to another.

The anatomical framework proposed here may also warrant evaluation in other spondyloarthritis-related conditions, although the present findings should not be directly extrapolated beyond PsA. Costovertebral and costotransverse structural involvement has been demonstrated on CT in axSpA, supporting the potential relevance of systematic posterior thoracic joint assessment in this population (8). PAO also frequently involves the anterior chest wall, particularly the sternoclavicular and sternocostal regions (21), suggesting that an anterior–posterior thoracic perspective may be of interest. However, the prevalence, lesion composition, and spatial distribution of structural abnormalities are likely to vary across disease entities. Similarly, because pSpA encompasses a heterogeneous group of disorders, the present PsA-derived phenotype definitions cannot be assumed to apply to non-psoriatic pSpA. Disease-specific external validation is therefore required before extending this framework to axSpA, PAO, or other forms of pSpA.

Several limitations constrain the interpretation of these results. First, the retrospective, single-center design and the use of clinically indicated chest CT introduce selection bias. Patients who underwent CT may not represent the broader PsA population. The absence of a non-PsA control group limits assessment of disease specificity. The “negative” phenotype is not a normal control group but consists of PsA patients without CT-positive involvement of the predefined thoracic joints. Future studies including matched controls are needed to confirm specificity. Second, the A–P group was older and had a longer disease duration, leaving the possibility of residual confounding from cumulative degeneration, prior disease activity, treatment exposure, or other unmeasured factors. PASI was incompletely and non-uniformly recorded and not systematically assessed within a standardized interval relative to baseline CT, while current cutaneous psoriasis status could not be reliably determined, precluding evaluation of its relationship with thoracic topographic phenotype. Third, the primary positivity rule included score-1 suspicious abnormalities and favored sensitivity over specificity. This decision may have affected both the phenotype distribution and the absence of anterior-only cases. Fourth, the total burden outcome was compositionally coupled to the A–P definition, although the posterior-burden analysis partly addressed this concern. Fifth, CT depicts structural change but not active synovitis, osteitis, or enthesitis, so the biological activity underlying each pattern could not be determined. Manubriosternal and sternocostal joints were also not assessed, meaning that the proposed ring does not encompass the entire anterior chest wall. Finally, follow-up examinations were sparse and non-standardized. The exploratory progression counts should therefore not be interpreted as evidence of prognosis or treatment response.

## Conclusion

In conclusion, this study suggests that CT-detected structural involvement of the sternoclavicular, costovertebral, and costotransverse joints in psoriatic arthritis can be categorized into thoracic ring topographic phenotypes with distinct burden profiles, with combined anterior–posterior involvement indicating greater overall structural damage than posterior-only disease and supporting a spatial framework that may complement conventional site-based assessment and quantitative burden scoring.

## Data Availability

The original contributions presented in the study are included in the article/Supplementary Material, further inquiries can be directed to the corresponding author.
