# Supplementary material for: Anterior–posterior topographic patterns of thoracic joint structural abnormalities on chest CT in psoriatic arthritis
Source: Front Med (Lausanne). 2026 Aug 26;13:1944735. doi: 10.3389/fmed.2026.1944735 (PMC13553842; doi:10.3389/fmed.2026.1944735)
Supplement: Supplementary file 1 [file Datasheet1.docx]

**Table S1. Chest CT acquisition and reconstruction parameters**

| **Parameter** | **GE Revolution CT** | **United Imaging 80-row CT** | **Siemens 64-row CT** |
| --- | --- | --- | --- |
| **Scanner hardware class** | Wide-detector multidetector CT; 16-cm z-axis detector coverage capability | 80-row multidetector CT platform | 64-row multidetector CT platform |
| **Patient position** | Supine; arms raised when feasible | Supine; arms raised when feasible | Supine; arms raised when feasible |
| **Scan coverage** | Thoracic inlet to costophrenic angles; SCJ, CVJ, and CTJ included | Thoracic inlet to costophrenic angles; SCJ, CVJ, and CTJ included | Thoracic inlet to costophrenic angles; SCJ, CVJ, and CTJ included |
| **Tube voltage protocol** | Routine chest protocol centered on 120 kVp; automatic kV selection allowed when available | Routine chest protocol centered on 120 kVp; automatic kV selection allowed when available | Routine chest protocol centered on 120 kVp; CARE kV/automatic kV selection allowed when available |
| **Tube current control** | Automatic exposure control (GE Smart mA/Auto mA); mA varied by patient habitus | Automatic exposure control; mA varied by patient habitus | Automatic exposure control (CARE Dose4D or equivalent); mA varied by patient habitus |
| **Detector/collimation setting** | Thin-section helical chest acquisition using Revolution CT detector configuration | Thin-section helical chest acquisition using 80-row detector configuration | Thin-section helical chest acquisition using 64-row detector configuration |
| **Rotation time setting** | Approximately 0.28-0.50 s according to chest protocol | Approximately 0.35-0.50 s according to chest protocol | Approximately 0.33-0.50 s according to chest protocol |
| **Pitch setting** | Approximately 0.8-1.2, protocol- and coverage-dependent | Approximately 0.8-1.2, protocol- and coverage-dependent | Approximately 0.8-1.2, protocol- and coverage-dependent |
| **Matrix / field of view** | 512 x 512; FOV adjusted to thoracic coverage and body habitus | 512 x 512; FOV adjusted to thoracic coverage and body habitus | 512 x 512; FOV adjusted to thoracic coverage and body habitus |
| **Routine image reconstruction** | Standard chest kernel with vendor-specific iterative reconstruction when used clinically | Standard chest kernel with vendor-specific iterative reconstruction when used clinically | Standard chest kernel with Siemens iterative reconstruction when used clinically |
| **Bone reconstruction kernel** | High-spatial-frequency bone/edge-enhancing kernel | High-spatial-frequency bone/sharp kernel | High-spatial-frequency bone kernel, such as B60f/B70f or equivalent |
| **Reconstructed section thickness** | 1.0 mm | 1.0 mm | 1.0 mm |
| **Reconstruction interval** | 1.0 mm or overlapping interval according to scanner protocol | 1.0 mm or overlapping interval according to scanner protocol | 1.0 mm or overlapping interval according to scanner protocol |
| **MPR review** | Axial, coronal, and sagittal 1.0-mm multiplanar reconstructions | Axial, coronal, and sagittal 1.0-mm multiplanar reconstructions | Axial, coronal, and sagittal 1.0-mm multiplanar reconstructions |
| **Window setting for scoring** | Bone window, WW/WL = 2000/500 HU | Bone window, WW/WL = 2000/500 HU | Bone window, WW/WL = 2000/500 HU |
| **Contrast enhancement** | Non-contrast or contrast-enhanced CT accepted; scoring used bone-window reconstructions | Non-contrast or contrast-enhanced CT accepted; scoring used bone-window reconstructions | Non-contrast or contrast-enhanced CT accepted; scoring used bone-window reconstructions |

**Note.** Values are presented as scanner-specific routine chest CT protocol settings or protocol ranges rather than fixed patient-level DICOM values. Tube current, pitch, and rotation time may vary between examinations because automatic exposure control and patient habitus were used in routine clinical scanning. For the present structural assessment, all target joints were reviewed on 1.0-mm thin-section multiplanar reconstructions using a bone window setting of WW/WL 2000/500 HU. SCJ, sternoclavicular joint; CVJ, costovertebral joint; CTJ, costotransverse joint; MPR, multiplanar reconstruction; WW, window width; WL, window level; HU, Hounsfield units.

**Table S2. Inter-reader reliability of CT structural features, severity scores, and phenotype classification**

| **Variable** | **N** | **Reader 1** | **Reader 2** | **Consensus** | **Agreement, %** | **Reliability estimate** | **95% CI** |
| --- | --- | --- | --- | --- | --- | --- | --- |
| **A. Binary CT structural features** | | | | | | | |
| SCJ erosion | 279 | 121 (43.4%) | 124 (44.4%) | 123 (44.1%) | 96.1 | κ = 0.920 | 0.874–0.966 |
| SCJ joint-space narrowing | 279 | 13 (4.7%) | 15 (5.4%) | 14 (5.0%) | 98.6 | κ = 0.850 | 0.705–0.994 |
| SCJ osteophyte | 279 | 24 (8.6%) | 26 (9.3%) | 25 (9.0%) | 97.8 | κ = 0.868 | 0.765–0.972 |
| SCJ sclerosis | 279 | 12 (4.3%) | 14 (5.0%) | 13 (4.7%) | 98.6 | κ = 0.839 | 0.684–0.994 |
| SCJ ankylosis | 279 | 5 (1.8%) | 6 (2.2%) | 5 (1.8%) | 99.6 | κ = 0.907 | 0.727–1.000 |
| CVJ erosion | 279 | 81 (29.0%) | 84 (30.1%) | 83 (29.7%) | 96.1 | κ = 0.905 | 0.851–0.960 |
| CVJ joint-space narrowing | 279 | 27 (9.7%) | 29 (10.4%) | 28 (10.0%) | 97.8 | κ = 0.881 | 0.787–0.975 |
| CVJ osteophyte | 279 | 218 (78.1%) | 221 (79.2%) | 220 (78.9%) | 96.8 | κ = 0.904 | 0.842–0.966 |
| CVJ sclerosis | 279 | 26 (9.3%) | 28 (10.0%) | 27 (9.7%) | 97.8 | κ = 0.877 | 0.780–0.974 |
| CVJ ankylosis | 279 | 9 (3.2%) | 11 (3.9%) | 10 (3.6%) | 98.6 | κ = 0.793 | 0.595–0.990 |
| CTJ erosion | 279 | 87 (31.2%) | 90 (32.3%) | 88 (31.5%) | 96.1 | κ = 0.909 | 0.856–0.962 |
| CTJ joint-space narrowing | 279 | 83 (29.7%) | 86 (30.8%) | 84 (30.1%) | 96.1 | κ = 0.907 | 0.853–0.961 |
| CTJ osteophyte | 279 | 223 (79.9%) | 226 (81.0%) | 225 (80.6%) | 96.1 | κ = 0.875 | 0.802–0.947 |
| CTJ sclerosis | 279 | 58 (20.8%) | 61 (21.9%) | 59 (21.1%) | 96.8 | κ = 0.904 | 0.842–0.966 |
| CTJ ankylosis | 279 | 68 (24.4%) | 71 (25.4%) | 70 (25.1%) | 96.8 | κ = 0.914 | 0.858–0.969 |
| **B. Patient-level structural burden scores** | | | | | | | |
| SCJ structural burden score | 279 | 2.0 ± 2.4 | 2.0 ± 2.3 | 0.0 (0.0–4.0) | — | ICC = 0.935 | 0.916–0.949 |
| CVJ structural burden score | 279 | 25.1 ± 17.8 | 25.3 ± 17.4 | 26.0 (12.0–34.0) | — | ICC = 0.970 | 0.963–0.976 |
| CTJ structural burden score | 279 | 18.9 ± 13.7 | 19.3 ± 13.9 | 18.0 (10.0–26.0) | — | ICC = 0.950 | 0.936–0.962 |
| Posterior structural burden score | 279 | 44.0 ± 25.7 | 44.5 ± 25.5 | 44.0 (22.0–58.0) | — | ICC = 0.972 | 0.966–0.978 |
| Total structural burden score | 279 | 46.0 ± 25.9 | 46.5 ± 25.7 | 46.0 (24.0–61.0) | — | ICC = 0.972 | 0.965–0.978 |

**Notes:** For binary features, Reader 1 and Reader 2 results are positive counts with percentages. For burden scores, results are mean +/- standard deviation. Binary features and patient-level classifications were evaluated using Cohen's kappa; ordinal grades were evaluated using quadratic-weighted Cohen's kappa; burden scores were evaluated using a two-way random-effects, absolute-agreement, single-measure intraclass correlation coefficient.

**Abbreviations:** CI, confidence interval; CTJ, costotransverse joint; CVJ, costovertebral joint; ICC, intraclass correlation coefficient; SCJ, sternoclavicular joint.

**Table S3.** Exploratory follow-up outcomes by thoracic ring topographic phenotype

| **Topographic phenotype** | **Patients with follow-up, N** | **Follow-up interval, months, median (IQR)** | **Progression, n** | **No change, n** | **Improvement, n** | **Progression rate** |
| --- | --- | --- | --- | --- | --- | --- |
| Anterior-posterior ring type | 36 | 21.9 (13.1-33.6) | 10 | 26 | 0 | 27.8% |
| Posterior paravertebral-only type | 29 | 23.3 (12.8-35.2) | 4 | 24 | 1 | 13.8% |
| Negative type | 10 | 10.0 (8.1-24.4) | 0 | 10 | 0 | 0.0% |
| Overall | 75 | 21.9 (10.5-33.7) | 14 | 60 | 1 | 18.7% |

*Note.* Follow-up analyses are exploratory because only 75 patients had follow-up data and progression events were sparse. Values are n or n (%).

These results should not be interpreted as treatment-response or prognostic model endpoints.

**Table S4.** Ablation, strict-ankylosis sensitivity, and alternative-outcome analyses

| **Validation type** | **Outcome** | **Model/analysis** | **N** | **R²** | **Adjusted R²** | **AIC** | **BIC** | **Definition or interpretation** |
| --- | --- | --- | --- | --- | --- | --- | --- | --- |
| Ablation/model alternative | Total CT structural damage burden | Full single-joint model | 279 | 0.668 | 0.658 | 2306.7 | 2339.3 | SCJ+CVJ+CTJ |
| Ablation/model alternative | Total CT structural damage burden | SCJ removed | 279 | 0.658 | 0.649 | 2312.6 | 2341.6 | Tests the added value of the anterior chest-wall endpoint |
| Ablation/model alternative | Total CT structural damage burden | CVJ removed | 279 | 0.632 | 0.623 | 2333.2 | 2362.3 | Tests the added value of CVJ |
| Ablation/model alternative | Total CT structural damage burden | CTJ removed | 279 | 0.660 | 0.651 | 2311.0 | 2340.1 | Tests the added value of CTJ |
| Ablation/model alternative | Total CT structural damage burden | Joint-count model | 279 | 0.650 | 0.643 | 2316.9 | 2342.3 | Keeps only the number of positive target joints |
| Ablation/model alternative | Total CT structural damage burden | Thoracic topographic model | 279 | 0.626 | 0.617 | 2337.6 | 2366.6 | Keeps the spatial combination pattern |
| Ablation/model alternative | Posterior structural damage burden | Full single-joint model | 279 | 0.649 | 0.639 | 2298.7 | 2331.3 | SCJ+CVJ+CTJ |
| Ablation/model alternative | Posterior structural damage burden | SCJ removed | 279 | 0.648 | 0.638 | 2298.0 | 2327.1 | Tests the added value of the anterior chest-wall endpoint |
| Ablation/model alternative | Posterior structural damage burden | CVJ removed | 279 | 0.610 | 0.600 | 2326.3 | 2355.3 | Tests the added value of CVJ |
| Ablation/model alternative | Posterior structural damage burden | CTJ removed | 279 | 0.641 | 0.632 | 2303.3 | 2332.4 | Tests the added value of CTJ |
| Ablation/model alternative | Posterior structural damage burden | Joint-count model | 279 | 0.617 | 0.609 | 2319.2 | 2344.7 | Keeps only the number of positive target joints |
| Ablation/model alternative | Posterior structural damage burden | Thoracic topographic model | 279 | 0.604 | 0.594 | 2330.7 | 2359.8 | Keeps the spatial combination pattern |
| Strict-ankylosis sensitivity | Topographic distribution | Strict anterior-posterior type | 2 | — | — | — | — | Positivity defined only by partial/complete bony ankylosis; strict SCJ ankylosis cases were too few for primary grouping |
| Strict-ankylosis sensitivity | Topographic distribution | Strict posterior-only type | 72 | — | — | — | — | Positivity defined only by partial/complete bony ankylosis; strict SCJ ankylosis cases were too few for primary grouping |
| Strict-ankylosis sensitivity | Topographic distribution | Strict isolated anterior chest wall type | 3 | — | — | — | — | Positivity defined only by partial/complete bony ankylosis; strict SCJ ankylosis cases were too few for primary grouping |
| Strict-ankylosis sensitivity | Topographic distribution | Strict no-ankylosis type | 202 | — | — | — | — | Positivity defined only by partial/complete bony ankylosis; strict SCJ ankylosis cases were too few for primary grouping |
| Alternative outcome | Total number of structural CT features | 2.0 (1.0–4.0) | 279 | — | — | — | — | Three-group Kruskal-Wallis P<0.001; anterior-posterior ring type: 3.0 (2.0–5.0), posterior paravertebral-only type: 1.0 (1.0–2.0), Negative type: 0.0 (0.0–0.0) |

*Note.* Ablation/model-alternative rows compare model fit after removing individual joint endpoints or replacing single-joint indicators with joint count or topographic phenotype.

Strict-ankylosis sensitivity defined positivity only by partial/complete bony ankylosis; strict SCJ ankylosis cases were too few for primary grouping.

The alternative outcome row summarizes the total number of structural CT features and reports Kruskal-Wallis testing across the three primary topographic phenotype groups.

**Table S5.** Comparative performance of clinical, single-joint, joint-count, and topographic models for structural damage burden

| **Outcome** | **Model** | **Main variables** | **N** | **R²** | **Adjusted R²** | **AIC** | **BIC** | **RMSE** | **Delta R² vs clinical** | **Delta AIC vs clinical** | **Interpretation** |
| --- | --- | --- | --- | --- | --- | --- | --- | --- | --- | --- | --- |
| Total CT structural damage burden | Clinical baseline model | Age + sex + disease duration + elevated CRP + elevated ESR | 279 | 0.515 | 0.506 | 2406.1 | 2427.9 | 17.67 | 0.000 | 0.0 | Reference model |
| Total CT structural damage burden | Single-joint model | Clinical variables + SCJ/CVJ/CTJ positivity | 279 | 0.668 | 0.658 | 2306.7 | 2339.3 | 14.62 | 0.153 | -99.4 | Best numerical fit; distinguish predictive fit from the anatomic interpretability of the topographic model |
| Total CT structural damage burden | Joint-count model | Clinical variables + number of positive target joints | 279 | 0.650 | 0.643 | 2316.9 | 2342.3 | 15.00 | 0.135 | -89.2 | Close to the single-joint model; simple joint count retained explanatory value |
| Total CT structural damage burden | Thoracic topographic model | Clinical variables + anterior-posterior/posterior-only/low-burden type | 279 | 0.626 | 0.617 | 2337.6 | 2366.6 | 15.51 | 0.111 | -68.5 | Improved over the clinical baseline, but AIC/R² did not exceed the single-joint model in the current data |
| Posterior structural damage burden | Clinical baseline model | Age + sex + disease duration + elevated CRP + elevated ESR | 279 | 0.500 | 0.491 | 2391.7 | 2413.5 | 17.21 | 0.000 | 0.0 | Reference model |
| Posterior structural damage burden | Single-joint model | Clinical variables + SCJ/CVJ/CTJ positivity | 279 | 0.649 | 0.639 | 2298.7 | 2331.3 | 14.41 | 0.149 | -93.0 | Best numerical fit; distinguish predictive fit from the anatomic interpretability of the topographic model |
| Posterior structural damage burden | Joint-count model | Clinical variables + number of positive target joints | 279 | 0.617 | 0.609 | 2319.2 | 2344.7 | 15.06 | 0.117 | -72.5 | Close to the single-joint model; simple joint count retained explanatory value |
| Posterior structural damage burden | Thoracic topographic model | Clinical variables + anterior-posterior/posterior-only/low-burden type | 279 | 0.604 | 0.594 | 2330.7 | 2359.8 | 15.32 | 0.104 | -61.0 | Improved over the clinical baseline, but AIC/R² did not exceed the single-joint model in the current data; report this transparently |

*Note.* The clinical baseline model included age, sex, disease duration, elevated CRP, and elevated ESR. Delta R² and Delta AIC are relative to the clinical baseline model for the same outcome.

Lower AIC, lower BIC, lower RMSE, and higher R² indicate better numerical fit. The topographic model should be interpreted as an anatomic explanatory framework rather than as outperforming all alternative predictive models in the current dataset.

AIC, Akaike information criterion; BIC, Bayesian information criterion; CRP, C-reactive protein; ESR, erythrocyte sedimentation rate; RMSE, root mean square error.

**Figure S1. Representative CT findings of structural damage by severity.**
CT images show structural damage grades 1–4 in the (A) sternoclavicular joint (SCJ), (B) costotransverse joint (CTJ), and (C) costovertebral joint (CVJ). Grades 1–4 indicate suspicious, mild, moderate, and ankylotic changes, respectively.
